# Machine-Learning-Based Real-Time Multi-Camera Vehicle Tracking and Travel-Time Estimation

**DOI:** 10.3390/jimaging8040101

**Published:** 2022-04-06

**Authors:** Xiaohui Huang, Pan He, Anand Rangarajan, Sanjay Ranka

**Affiliations:** Department of Computer and Information Science and Engineering, University of Florida, 432 Newell Drive, CSE Building, Gainesville, FL 32611, USA; hxhcindy@gmail.com (X.H.); pan.he@ufl.edu (P.H.); anand@cise.ufl.edu (A.R.)

**Keywords:** vehicle signature, travel-time computation, intelligent transportation systems, deep learning

## Abstract

Travel-time estimation of traffic flow is an important problem with critical implications for traffic congestion analysis. We developed techniques for using intersection videos to identify vehicle trajectories across multiple cameras and analyze corridor travel time. Our approach consists of (1) multi-object single-camera tracking, (2) vehicle re-identification among different cameras, (3) multi-object multi-camera tracking, and (4) travel-time estimation. We evaluated the proposed framework on real intersections in Florida with pan and fisheye cameras. The experimental results demonstrate the viability and effectiveness of our method.

## 1. Introduction

Mitigating traffic congestion and improving safety are the important cornerstones of transportation for smart cities. With growing urbanization around the world, traffic congestion along high-volume signalized traffic corridors (arterials) is a major concern. Congestion negatively affects productivity, leading to loss of work hours, thus impacting the economy. Congestion also impacts the well-being of society and the environment [1,2].

One of the important measures of congestion is arterial travel time [3]. This value is the expected travel time that a vehicle will take to complete its journey along a signalized traffic corridor and is affected by many factors, such as traffic conditions and departure time. This quantity is easy to interpret by traffic engineers and city authorities as well as the general public. Traffic engineers can use travel time to identify problematic locations and timing problems, which can lead to improvement of the overall performance of a traffic system [4,5,6,7,8,9]. Current performance evaluations only include a limited comparison of before-and-after travel-time data to demonstrate the effectiveness of signal retiming [10] efforts.

Most existing research on travel-time estimation focuses on using non-vision sensors, such as GPS [11,12] and loop detectors [13]. Due to the simplicity of implementation and low computational cost, historical-data-based travel-time estimation methods have been widely used in practice [14,15]. Other approaches rely on machine learning and data mining on toll collection information [16], probe cars [17], highways [18], and trip datasets [19].

**Figure 1:**
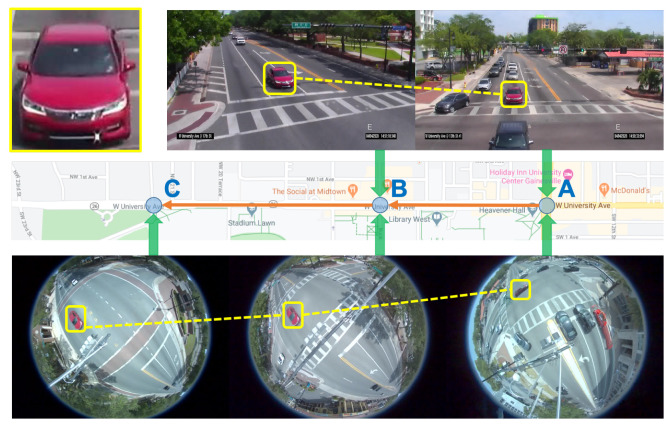
An illustration of multi-camera vehicle tracking and travel-time estimation problem. In this example, we have three intersections, A, B, and C, in a corridor. They all have one fisheye camera, and A and B have one additional pan camera. The multi-camera vehicle tracking and travel-time estimation problem seeks to solve: (1) Is the red car the same vehicle among these intersections? and (2) What is its arrival and departure time at each intersection?

However, traffic patterns vary dynamically throughout the day as well as globally within the network, and there is a need for continuous monitoring and evaluation of signal timing parameters based on performance and fluctuation demands. To achieve this, travel times have to be calculated at regular intervals. In addition, it is important to understand the distribution of travel times rather than average travel times because the tail of the distribution gives traffic engineers rich information. Actual travel time often has a multimodal distribution, and the expected values are not always sufficient. The advent of video cameras at traffic intersections has opened the possibility of using them for instantaneous travel-time computation and is the focus of this paper. The novelty of our travel-time estimation is twofold. First, we used car signatures to estimate vehicle departure and arrival time from video across multiple cameras. In addition, we leveraged signal phasing and timing (SPaT) data in this system. To the best of our knowledge, this represents the first end-to-end framework that estimates vehicle travel time on corridor intersections using multiple videos and SPaT data (Figure 1).

We achieved the above goals by accurately tracking a small subset of vehicles on a corridor or network. This, along with reasonably synchronized clocks at each intersection, will allow for accurate travel-time computation for that subset of cars and can be used for travel-time estimation. The latter is a relatively straightforward computation if tracking can be accurately achieved. It is worth noting that it is more important for the tracking to be accurate for a subset of cars compared to tracking a larger fraction of cars with less accuracy. That is because accurate tracking can provide good input for re-identifying cars across multiple cameras and thus compute a more accurate travel time.

We developed a unified system to track vehicles across multiple video cameras installed on corridor intersections. It uses a robust signature-matching algorithm to handle a variety of vehicles under different camera perspectives. This algorithm can efficiently identify the signatures of vehicles in real-time and further help the computation of accurate detection of arrival time and departure time at each intersection. At any signal, a subset of vehicles may have to wait when the light turns red. This, in general, creates additional delays for a subset of vehicles. Our approach can robustly tackle these challenges. Additionally, our approach leverages the fact that the ordering of vehicles from one intersection to another one does not change substantially. This is not a requirement for our approach to be effective, but this property can reduce the computation time required by the matching algorithm. Although much of the paper is described in terms of computing travel times on a corridor, the basic ideas are promising in the extension to work on a mesh of intersections.

In summary, we propose a real-time video processing system for multi-camera vehicle tracking and travel-time estimation with the following key contributions:

1. We propose a video-based signature re-identification method with high precision that plays a key role in multi-camera vehicle tracking.
2. We introduce a novel way to extend pairwise signature matching to phase-based signature matching to tackle the sequential group signature re-identification problem on video data.
3. We propose a novel method for vehicle travel-time estimation using arrival and departure information at each intersection.
4. We evaluate the proposed framework on a novel dataset from intersections in Florida with pan and fisheye cameras. The experimental results demonstrate promising performance for camera signature ReID and travel-time estimation.

## 2. Related Work

In this section, we present some relevant methods from three research topics: object-signature ReID and multi-object multi-camera tracking and travel-time estimation.

### 2.1. Object-Signature ReID

Most existing object-signature re-identification (ReID) methods focus on image datasets while paying less attention to the processing of large-scale video datasets [20]. These methods, together with the multi-object multi-camera tracking method, tend to neglect the temporal cues in traffic videos, such as phase (traffic light) information.

Image-based object ReID, particularly ReID of humans, has been studied for many years to extract reliable features [21], to design accurate feature distance metrics [22,23], and to deal with local spatial misalignment between people [24,25]. Similar approaches have appeared in the literature investigating the handling of objects with different types, such as cars [26], or to handle more complex scenes where many cameras may be present (a collection of [27] videos [28] or images [29,30]). Recent approaches have started to tackle the problem of single-person ReID via deep-learning models that are powerful in extracting rich features from images. Many works [31] have exploited the pairwise labels derived by sampling many pairs of positive and negative data while utilizing various convolutional neural network (CNN) architectures. One popular approach [21,32,33] is to learn a representative feature-embedding-space with the triple-loss objective function, which has shown superiority in boosting performance [34,35].

Up to now, most work has focused on ReID at the individual-level, whereas ReID at the group-level is heavily overlooked. Group ReID introduces new additional challenges in modeling due to the presence of significant group layout and membership changes. Though a previous approach [36,37] introduced the interaction of people or groups into the ReID process, their study was limited to only improving the performance of ReID for each person. It remains under-explored and requires further full modeling to incorporate the characteristics of groups.

Several methods have been proposed to solve the group ReID problem, with most of them aimed at performing group-by-group matching with global or semi-global functions. For example, based on the global statistics of appearance properties, [38] proposes a discriminative covariance descriptor for group images. Further, [39] obtains a global group representation by merging the sparse driver descriptions of all patches. The local interaction information in the group is not likely to be captured via global or semi-global functions, which limits the current methods when trying to handle complex scenes with significant variations in group appearance. Alternatively, a recent local-based approach [40] has performed group ReID by selecting corresponding patch pairs and conducting cross-view, group-image-based patch matching.

Most research on multi-camera-tracking and object ReID has focused on visual-similarity modeling of vehicles, neglecting sequence information of vehicle groups and phase information of traffic. It is non-trivial to generate video-based signatures of vehicles and do pair-wise and phase-based matching across different camera views. The key component of our signature ReID is discriminative ReID learning based on the popular ResNet-50 [41]. The novelty of our object-signature ReID is that we have extended image-based pair-wise signature ReID to phase-based signature ReID with signal timing constraints to tackle the sequential group ReID problem. It is an efficient method to do signature group alignment. We consider the challenges when applying signature ReID to real-world traffic data, and we define five types of signature-matching scenarios. We evaluate our method on the novel dataset collected from pan and fisheye cameras installed in Gainesville, FL, USA.

### 2.2. Multi-Camera Multi-Object Tracking

This has received increasing research interest, as demonstrated in [42]. One critical step is to capture the spatial relations between cameras, which could be obtained by tracking known identities to learn an explicit mapping in 3D [4,5,6,43,44] or by examining entry/exit rates across multiple cameras [8,9,45]. While several methods are based on completely overlapping and unobstructed views [46,47,48,49,50], a pre-processing step could potentially be integrated to fuse data when partially overlapping views are presented. Both entry points and exit points of a person can be explicitly modeled on the ground [6,8,9,45] or image plane [4,7].

Correlation-filter-based trackers have achieved promising performances in recent advances. This type of tracker leverages online-learned correlation filters to find the target location and determine the target scale using a fixed scale factor. One of the representatives is KCFs [51]. The advantage of this type of tracker is fast-tracking speed due to transforming the time-domain calculations into frequency-domain operations. However, for objects with fast movement or large shape changes, it leads to learning more background information.

Some of the prior methods are either end-to-end or achieving real-time performance, as these two factors play an important role in large-scale, big-data transportation applications. We build our multi-camera vehicle tracking and travel-time estimation system upon our real-time single-camera tracking framework mainly by introducing video-based signature ReID. The advantage of our system is that it is end-to-end, real-time, and scalable.

### 2.3. Travel-Time Estimation

Knowledge about travel time is very important to traffic management and operation—it could not only help individuals in making informed and efficient travel decisions but also traffic agencies in identifying problematic locations and taking proper measures for traffic control, which improves the overall performance of a traffic system [4,5,6,7,8,9,44].

However, most research on travel-time estimation has focused on modeling based on data from non-vision sensors, such as GPS and loop detectors, while methods leveraging video data from intersection cameras are seldom studied. First attempts at travel-time estimation relied on historical data [14,15], which is still widely used in practice due to the simplicity of implementation and low computational cost. The second strategy is parametric methods, often using mathematical equations [52]. The robustness of these two methods is limited. The third approach uses machine learning, data mining [16,17], or hybrid power [18,19] to discover patterns and knowledge in data. However, these methods are limited to a certain type of sensors data (GPS [11,12], loop detectors, etc.), excluding vision sensors data.

The key component of our signature ReID is discriminative ReID learning based on the popular ResNet-50 [41]. The novelty of our object-signature ReID is that we have extended image-based, pairwise-signature ReID to phase-based signature ReID with signal timing constraints to tackle the sequential group ReID problem. It is an efficient method for signature group alignment. We consider the challenges when applying signature ReID to real-world traffic data, and we define five types of signature-matching scenarios. We evaluate our method on the novel dataset collected from multiple pan and fisheye cameras installed in Gainesville, FL, USA. We show that our system is end-to-end, real-time, and scalable.

## 3. Methodology

The goal of multi-object multi-camera tracking is to detect and track multiple objects within one camera (tracklets) and further perform multiple-camera tracklet matching to derive trajectories of vehicles. The arrival and departure timestamps of the vehicles are then utilized to estimate travel-time distribution. The input is the traffic video data collected from multiple intersections. For the sake of simplicity, we assume a three-camera system with one camera for each of three successive intersections (corridor intersections). It is easy to generalize these ideas to a larger number of intersections, e.g., mesh intersections. We assume that each intersection can have one or more cameras, potentially of different types. In particular, the corridor that we experimented with has three intersections: labeled A, B, and C. Both A and B have one fisheye camera and one pan camera, while C only has a fisheye camera. Thus, this corridor corresponds to two tracking channels: (1) in the fisheye channel, the inputs are video sequences from three fisheye cameras; and (2) in the pan channel, the inputs are video sequences from two pan cameras.

**Figure 2:**
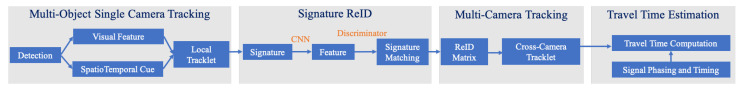
An illustration of the pipeline for the proposed method. Given intersection videos as input, a single-camera tracker first detects vehicles and generates local tracklets. Then a ReID discriminator computes matching among vehicles under the constraints of phase information. Finally, we apply a merging algorithm to update tracking results for multi-camera tracking and compute the travel time of each vehicle using the timestamp information.

Our proposed method contains four major modules, as shown in Figure 2:

1. Multi-object single-camera tracking module: we use tracking-by-detection based methods to track vehicles (car, bus, and truck) in every single camera and generate local tracklets.
2. Video-based signature ReID module: We parse our local tracklets into signatures in different cameras with departure timestamps and use a deep-learning-based discriminative model to match the local tracklets across multiple cameras.
3. Vehicle tracking module: we leverage the signature-matching results to associate the local tracklets (full timestamps) across multiple cameras by tracklet-to-signature assignment. By matching the separate local tracklets among multiple cameras, we generate a complete trajectory for each vehicle across all cameras in multiple road segments.
4. Travel-time-estimation module: we compute travel time based on arrival and departure timestamps from final across-camera tracking results. Our assumption is that the input videos are collected at nearly the same time and that local clock time can be converted to a common global time.

Additionally, before utilizing the second video-based-signature ReID module, we can filter out tracklets using trajectory direction detection (go-straight, turn-left, or turn-right) and only focus on one direction (e.g., go-straight traffic flow from camera A to camera B then camera C).

### 3.1. Multi-Object Single-Camera Tracking

We used a deep learning model to detect and track road objects, calculate speed after deformation correction, and then calculate the map-based trajectory. The deep object detector trained on fisheye video samples is based on the architecture of YOLO [53]. According to the intersection attributes, we specify five object categories: pedestrians, motorcycles, cars, buses, and trucks.

The multi-object tracker is built on DeepSort [54] and uses the conventional single-hypothesis tracking method, with recursive Kalman filtering and frame-by-frame data association. However, when the intersection becomes crowded or large buses or trucks appear, there is an occlusion problem. Therefore, some road objects can obtain new recognition after the occlusion disappears, which forces us to integrate object signatures or object ReID features.

**Figure 3:**
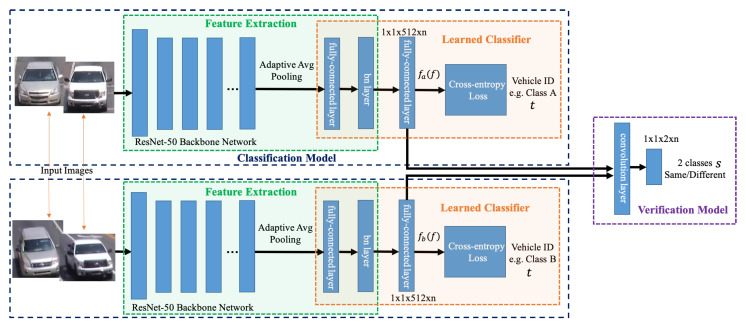
An illustration of the network architecture of our proposed two-loss signature ReID model using ResNet-50 as backbone.

**Figure 4:**
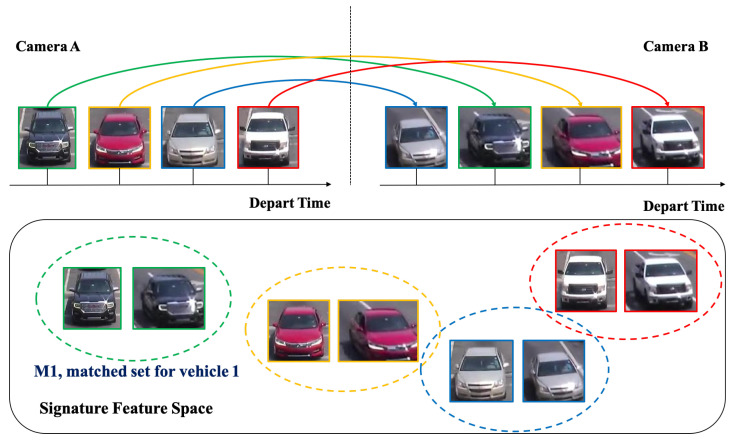
An illustration of matched-vehicle sets and their distributions in the feature space. The colored solid arrows indicate the one-to-one mapping results.

**Figure 5:**
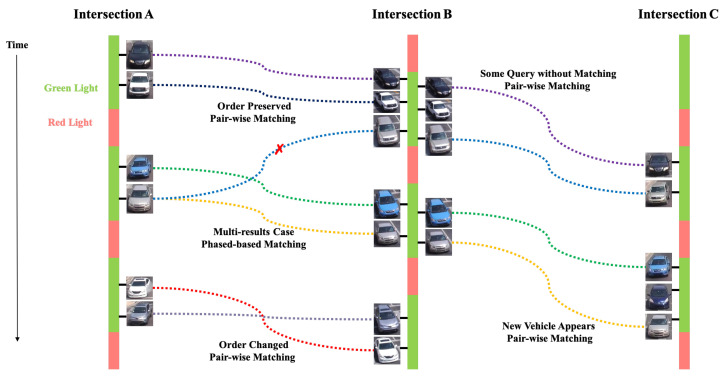
An illustration of across-camera matching. Our primary matching strategy is pair-wised matching using our discriminator. For cases with multiple matching results, phase information is used as an additional constraint to refine matching. We defined five types of matching cases: (1) order preserved (pairwise matching)—order of signature sequence does not change among intersections; (2) multi-results (phased-based matching); (3) order changed (pairwise matching); (4) some query without matching (pair-wise matching); and (5) new vehicle appears (pairwise matching).

We introduce a deep cosine metric learning component to learn the cosine distance between road objects and integrate it as the second metric for the association problem in multi-object tracking [55,56]. The cosine distance includes the appearance information of the road object to provide useful hints to restore the identity when the discriminative power of the motion feature is small. We chose the cosine metric for measuring feature similarity because it naturally conducts feature vector normalization and its range is bounded. We train a deep cosine metric learning model on the VeRi dataset [20]. To ensure we generate good inputs for ReID, we introduce a track-direction-detection component to focus on vehicles that would go straight from camera A to camera B then camera C. Figure 3 shows the overall model architecture of our signature ReID network. Figure 4 shows the matched-vehicle sets and their distributions in the feature space. The direction detection is based on a computation of vehicle velocity direction from trajectory data. The SPaT data is used before ReID to group local tracklets. Figure 5 is an illustration of across-camera matching. We divide tracks into different phases based on green-light timestamps. The motivation for using signal phase info is based on the assumption that vehicle groups in one phase (stopped at interaction A for red light) are more likely to arrive at the next intersection B within one phase. So a two-phase or three-phase selection pool is reasonable for ReID windows. We leverage phase information to make a coarse-to-fine matching strategy to (1) save query cost and (2) improve matching performance a little bit for corner cases. However, signal-phase information is not a requirement for using our system.

### 3.2. Pairwise Signature ReID

Vehicle signature ReID is used to determine whether there is a specific vehicle across images or video frames from non-overlapping cameras. In real-world scenarios, due to the differences between different cameras setting (camera type, perspectives, height, FOV, etc.), vehicles have both rigid and flexible characteristics. Their appearance has constraints within the range of color, size, and type, and the appearance is easily affected by occlusion and viewing angle, which makes vehicle ReID a challenging task.

The popular deep learning ReID methods are leveraging high-level semantic information and designing novel objective functions to encourage models to learn a discriminative feature representation. The two main types of loss functions are classification loss and verification loss. Classification loss directly uses the label information of the signature to do multi-class classification. Verification loss determines whether the two input signatures belong to the same object. Hybrid approaches have shown improved performance by combining both in model learning [57]. Therefore, we chose to use both the classification loss and the verification loss.

#### 3.2.1. Overall Network

Our signature ReID model uses deep learning models to obtain discriminative feature representation. Figure 3 shows the overall model architecture of our signature ReID network. Our network consists of two sub models: the classification model and the verification model. Given vehicles image as input, the classification model distinguishes images by dividing them into an N class based on which type of vehicle they are, which is learned by a softmax loss. For example, during training we assume a total of N objects presented in the training dataset, we therefore use an N-class softmax classifier to distinguish these objects. However, when moving to inference, the objects are completely changed, thus the N-class classifier is not longer useful. As our main goal is to obtain the signature of each object rather than an actual classification, we instead use the features of the last fully-connected layer (a feature vector of 512) as its signature feature, before the softmax classifier. This makes the model applicable to both training and inference for any object.

Given a pair of vehicle images, the verification model determines whether the two images come from the same object by mixing their corresponding 512-dim embedding features via element-wise multiplication and stacking several convolutional layers to form one binary classifier. We use the 512-dim fully-connected feature as vehicle signature descriptor. If two vehicle images come from the same vehicle (having the same class ID), we classify them as 1, otherwise 0. The binary classification will encourage the 512-dim signature features to be similar to each other if belonging to the same object and to be far if coming from different objects.

Since signature ReID is the key to multi-camera tracking, it is very important that our ReID discriminator achieves high precision to find the best signature matching. Given a pair of images, we apply a Siamese network to calculate the classification losses for both images as well as to compute the verification loss between the two. It predicts the IDs and the similarity score of two images which supervised by the classification and the verification labels.

#### 3.2.2. Classification Loss

In the Siamese architecture, the two ResNet-50 models share weights in the network framework and predict the category labels of the pair image. The fully connected layers and the last pooling layer of the original model are removed. We use the adaptive average pooling to obtain a feature with a fixed dimension, followed by several new fully-connected and batch norm layers. Before feeding to the final classifier, we are able to obtain a 512-dim fully connected feature (denoted as *f*), which serves as the vehicle signature descriptor. Because we train the model on the vehicle ReID dataset VeRi [20] (with 576 vehicles—each with multiple images), the classifier consists of one fully connected layer with 576 dimensions (denoted as $\phi_{\theta }$ with parameters θ) and a softmax layer to obtain the class distribution. The cross-entropy loss, which is denoted as follows, is used for training the classifier:

(1) $$\begin{array}{cc} \hat{p} & =softmax(\phi_{\theta }\left(f\right)), \end{array}$$

(2) $$\begin{array}{cc} \mathrm{Classif}(f,t,\phi_{\theta }) & =\sum_{i=1}^{K}-p_{i}\log\left({\hat{p}}_{i}\right) \end{array}$$

where *t* is the target class, $\hat{p}$ is the predicted probability, and $p_{i}$ is the target probability. *K* is the total number of classes and is set to 576 in our case.

#### 3.2.3. Verification Loss

The verification loss directly takes two descriptor vectors $f_{1}$ and $f_{2}$ computed from two images as inputs. We compute $f_{s}=f_{1}*f_{2}$. After this, we stack several fully-connected layers and add the softmax classifier to project $f_{s}$ to a 2-dim feature vector, representing the predicted probability of whether the two images come from the same vehicle or not. It therefore regards the ReID problem as a binary classification problem where the cross-entropy loss function is used similar to the classification model. The cross-entropy loss in the verification loss is as follows:

(3) $$\begin{array}{cc} \hat{q} & =softmax(\gamma_{\theta }\left(f_{s}\right)), \end{array}$$

(4) $$\begin{array}{cc} \mathrm{Verif}(f_{1},f_{2},s,\gamma_{\theta }) & =\sum_{i=1}^{2}-q_{i}\log\left({\hat{q}}_{i}\right) \end{array}$$

where $f_{1}$ and $f_{2}$ are two tensors of size 1×1×512, *s* is the target category (indicating same or different target), and $\gamma_{\theta }$ denotes the whole operation that maps $f_{s}$ to the 2-dim feature vector before the softmax layer.

#### 3.2.4. Discussion of the Losses

The verification loss is directly trained on the similarity between two features through an intuitive pairwise comparison method. The disadvantage of the verification loss is that only the verification result is considered in the training, while the annotation information is not fully utilized. Also, the association information between the image pairs and other images in the data set is not utilized.

The classification loss regards the vehicle ReID task as a classification task; each vehicle signature with the same identity is regarded as a category; and it learns directly from the input image and its identity ID. The inputs of the classification model are independent of each other, but a potential association relationship is implied because each signature has an implicit relationship with a signature with the same identity and a signature with different identities through category tags. The drawback in the classification model is that the training target is completely different from the test process. During the test, the embedding features are extracted and used to compute the similarity. However, the similarity measurement information between the image pairs is not considered at all during the training process.

**Figure 6:**
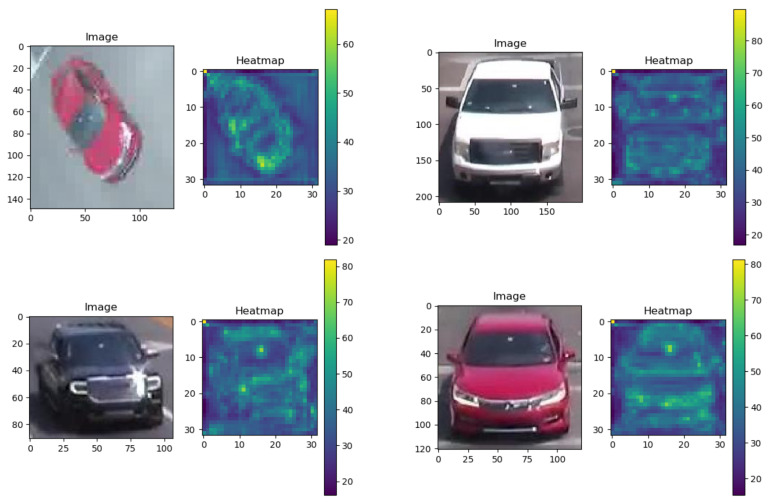
Samples of activation map of our model trained by the two losses.

We visualized the sum of several activation maps. The classification and verification network shows different activation patterns for vehicles. If only one loss is used, the network often finds a discriminatory part. Our proposed model takes advantage of the two networks, and the new activation map is mainly a combination of two separate maps. The proposed model enables more neurons to be activated. Figure 6 shows the two-dimensional visualization of the embedding.

#### 3.2.5. Training and Optimization

During training, we resize our input images to 256×128, shuffle the dataset, and use a random order of images. Then, we sample another image from the same (or different) classification ID to form a positive (or negative) pair. Initially, the ratio of negative pair and positive pair is 1:1. To reduce the forecast bias, we gradually multiply the ratio until it reaches 1:4. This is beneficial because the number of positive pairs is very limited, and the network has the risk of overfitting. We set the total training epochs to 75 and a batch size of 32. The training begins with an initial learning rate of 0.001, which is decayed to 0.0001 in the last five epochs. Stochastic gradient descent (SGD) is used to update network parameters. The weight of verification loss is set to 1, and the weight of two classification losses is set to 0.5. The dropout layers are applied as well.

In the testing phase, given a 256×128 image, we feed it our trained network and obtain the vehicle descriptor *f*. We obtain another $f_{\mathrm{flip}}$ by feeding its horizontally flipped image. Then, *f* and $f_{\mathrm{flip}}$ are averaged to form the final descriptor. After obtaining the descriptor of the candidate set (gallery set), we save it offline. For the query image, the descriptor is extracted instantly and calculated with the features of the candidate set (query set) to obtain the final matching result.

### 3.3. Multi-Camera Vehicle Tracking

We propose an effective and fast multi-camera vehicle-tracking strategy that can speed up the matching process and obtain more stable synchronization results by using intersection and other information. The SPaT data is used before ReID to group local tracklets, i.e. dividing tracks into different phases based on green-light timestamps.

Based on signature ReID features and temporal cues, we build our track descriptors with a bag of information: signature_match, camera_ID, timestamp, original_track_ID, and class (car, bus and etc.). We first compute the distance matrix using track descriptors as follows:

(5) $$distance_{N}={\left[d_{i,j}\right]}_{i,j=0}^{i,j=N}=1-cos\left(t_{i},t_{j}\right)$$

where $t_{i}$ and $t_{j}$ are track descriptors and *N* is total number of tracks of single camera tracking from all intersections. For vehicle in same cameras, We set a very high value in distance matrix since they are not supposed to be merged. We use one pre-defined thresholds for tracks merging.

Given the distance matrix $distance_{N}$, we update the previous multiple-object-tracking result *T* following these rules:

1. Sort the small traces according to their camera IDs, and compare only small traces with adjacent camera IDs.
2. If the minimum distance between the query track descriptor and all other track descriptors under different cameras is greater than the merging threshold, the tracklet $T_{i}$ is removed.
3. If the distance between $q_{i}$ and $T_{i}$ and $q_{j}$ and $T_{j}$ are all smaller than a predefined merging threshold, update the tracking ID of $T_{i}$ and $T_{j}$ to the same.

$T_{i}$ represents a gallery of small tracks with tracking function id *i* and $q_{i}$ represents small tracking queries with tracking IDs *i*. At intersections where each road segment is calculated and the data provided, vehicles can go multiple directions. It is more efficient to first detect trajectory direction and then apply ReID and multi-camera tracking. In our dataset, we have three intersections, A, B, and C, in a corridor. They all have one fisheye camera, and A and B have one additional pan camera. Since the three intersections form two line segments, the traffic flow we can monitor is bi-direction between 1 (camera A and camera B) and 2 (camera B and camera C). We filter out tracklets using trajectory direction detection (go-straight, turn-left, or turn-right) and focus on bi-directions (e.g., traffic flow go-straight from camera A to camera B then camera C or reversely). For intersection networks more complicated than line segments, our approach will also work for multiple directions, such as turn left and turn-right.

Without loss of generalization, for the rest of this paper, we focus on one-direction tracking for vehicles as they appear at intersection A, then intersection B, and then intersection C. We apply a simple direction-detection method based on trajectory information and intersection topology.

### 3.4. Travel Time Estimation

We assume that:

1. The clocks used for each of the input videos are already synchronized. If they are not, it is easy to add time corrections to our calculations;
2. We filter out tracklets using trajectory-direction detection (go-straight, turn-left, or turn-right) and only focus on one direction (e.g., go-straight traffic flow from camera A to camera B then camera C).

Since the videos are synchronized, we can compute travel time based on arrival and departure timestamps from final across-camera tracking results:

(6) $$T_{camera_{A},camera_{B}}V_{i}=timestamp_{B}V_{i}-timestamp_{A}V_{i}.$$

We define the departure timestamp as the first frame of the detected car passing the stop line of intersection as A and the arrival timestamp as the first frame of the detected car passing the stop line of intersection B. We did not use the first frame of camera A to eliminate bias when vehicles are waiting for the red light after camera A. In the overall pipeline, the single camera tracking generates tracklet results with information such as camera ID, video_start_time, frame ID, track ID, class, width, height, x, and y. The SPaT data provide phase information (red or green light) that we use to group a sub-sequence of tracklets to one phase. So for signature ReID, the input is signature representation that includes image (cropped from detection bounding boxes), track ID, camera ID, frame (departure time), class, and phase ID.

The pairwise matching windows are about two phases that usually contain 4 min video data with about 20 to 30 vehicles for one direction per camera. The ReID model outputs the matching results under constraints that (1) similarity score is higher than threshold, (2) two signatures are from different camera IDs, and (3) if multiple matching is computed, compute the phase-matching matrix (matching phase ID for all signatures in the same phase) and add a penalty for the matching results whose matrix distance is larger. We obtain the matching results and use them as registration information to do across-camera tracking for multiple local tracklets. Finally, we have multiple-camera tracking results with information to compute travel time: start intersection, end intersection, signature ID, departure_time, and arrival_time. We also implemented a visualization part of travel-time distribution to aid travel-time analysis.

## 4. Experiments

In this section, we first introduce our dataset and experiment settings. Then, we present qualitative and quantitative experimental results.

### 4.1. Experimental Setup and Parameter Setting

The experiments are performed on a 256 GB RAM machine with 16 CPU cores and one NVIDIA graphics card (Titian V). Our signature ReID is implemented on Pytorch. We train a ResNet-50-based signature discriminator on the VeRi dataset [20] as the pretrained model and evaluate on our dataset collected from pan and fisheye cameras of corridor intersections in Florida. The VeRi dataset contains over 50,000 images of 575 vehicles captured by 20 cameras. The threshold we set for the signature discriminator is 0.7.

### 4.2. Dataset

The data we used in this method are pure traffic video data collected from three intersections in a corridor in Gainesville, FL, USA. We refer to the three intersections as A, B, and C. Both A and B have one fisheye camera and one pan camera, while C only has one fisheye camera. The input of multi-object multi-camera tracking is the M video sequence from M cameras. Since we have two types of cameras, we have two tracking channels: (1) in the fisheye channel, the input is three video sequences from three fisheye cameras, and (2) in the pan channel, the input is two video sequences from two pan cameras. We evaluate our method on a self-curated video dataset collected from intersections in Gainesville, FL, USA. The resolution of pan camera video files is 1280×720 and that of the fisheye camera video files is 1280×960. The duration of the video files is about 16 to 20 min, containing approximately 8 or 9 phases. Our test set contains over 200 image sets with over 2500 vehicle detections. The vehicle types of the test set include car and bus. Based on single-camera tracking results, the signature of each vehicle includes the detection image (cropped from bounding boxes), camera ID, frame ID, and track ID. Each vehicle has about 11 images (near departure time) per camera, and we can apply multi-query matching setting by averaging these 11 images for signature ReID.

### 4.3. Qualitative Results

**Figure 7:**
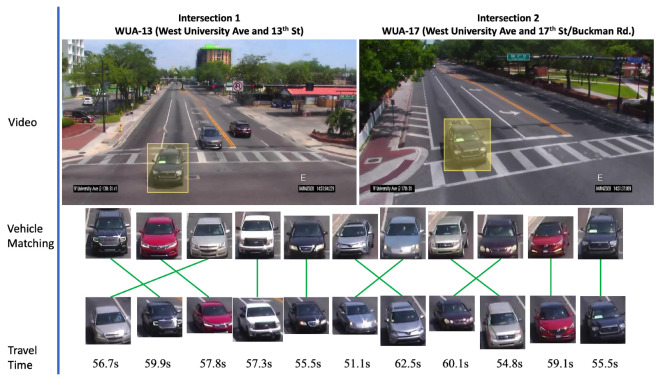
Multi-camera tracking and travel-time estimation results. Results of one-phase data after first-order matching, sequence-order checking, and travel-time estimation. Multi-matching did not happen, but sequence order changes did. The travel time between these two intersection ranged from 54 to 62 s.

**Figure 8:**
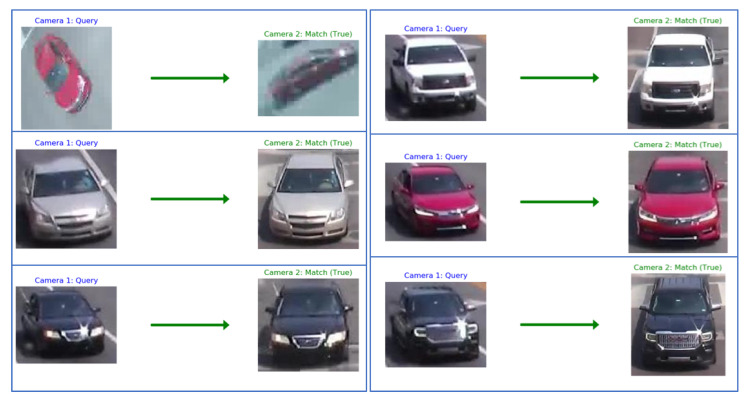
Samples of pairwise signature-matching results.

**Figure 9:**
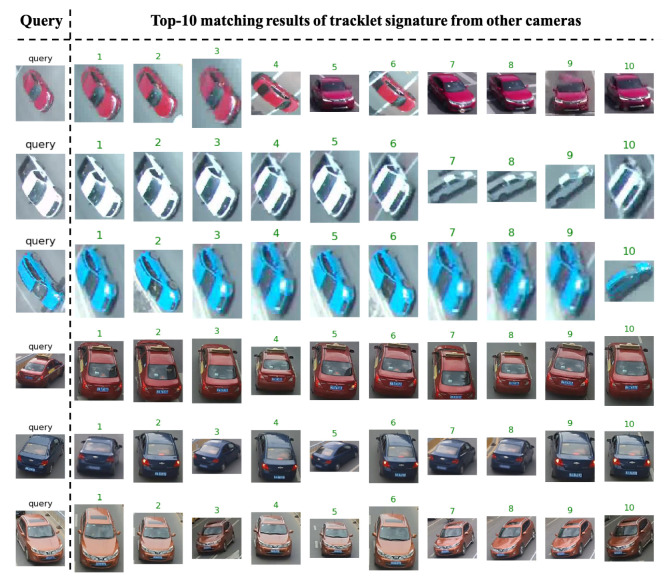
Samples of top 10 matching results of signature ReID on our dataset; (top 3 rows: pan+fisheye cameras) and Veri dataset [20] (bottom 3 rows).

We present qualitative results of signature matching. First, we present visualization of several activation maps of features from our signature-matching network. As shown in Figure 6, the signature ReID network shows different activation patterns for the vehicles. In Figure 7, we present sample results of cross-camera tracking with estimated travel time. In this example, all vehicles that are going straight (left two lanes) in intersection 1 have correct signature-matching from intersection 2. During this phase for the group of vehicles (11 vehicles), the average travel time is about 57.3 s. We present examples of pairwise signature-matching results in Figure 8, which demonstrates good matching for both fisheye camera and pan camera data. In Figure 9, we show examples of the top 10 matching results for queries from our dataset and the VeRi dataset. To compute the top 10 matching results, our gallery dataset includes both pan and fisheye cameras. The experimental results show that our discriminator network has reasonably high accuracy to retrieve correct matching.

### 4.4. Quantitative Results

To evaluate our signature ReID network, we first evaluate our test dataset with accuracy in terms of Rank-1, Rank-5, Rank-10, and mean average precision (mAP). The definition of mAP is as follows:

(7) $$mAP=\frac{1}{N}\sum_{i=1}^{N}\sum_{j=1}^{K_{q_{i}}}\left({\hat{r}}_{j}/r_{j}\right)$$

where query dataset denotes $Q=\left\{q_{1},q_{2},\cdots ,q_{i},\cdots ,q_{N}\right\}$ with *N* images and gallery dataset is denoted as $G=\left\{g_{1},g_{2},\cdots ,g_{i},\cdots ,g_{M}\right\}$ with *M* images. For each query $q_{i}$, we sort the gallery data in ascending order of ReID distance and denote sorted gallery as $G_{q_{i}}$ and the matching subset in $G_{q_{i}}$ is ${\hat{G}}_{q_{i}}=\left\{{\hat{g}}_{2},{\hat{g}}_{1},\cdots ,{\hat{g}}_{j},\cdots ,g_{{\hat{K}}_{q_{i}}}\right\}$. Assume ${\hat{g}}_{j}$ in $G_{q_{i}}$ has index $r_{j}$ and in ${\hat{G}}_{q_{i}}$ has index ${\hat{r}}_{j}$; we repeat this query processing for all query data. Rank-1 is defined as $rank1=\hat{N}/N$ where $q_{i}$ meets Rank-1 if the first match is correct in $G_{s}$ of $q_{i}$. The number of queries that meet Rank-1 is *Q* is *N*.

We train our signature ReID model on 576 training sets from the VeRi dataset and test it on our dataset with 200 testing sets (including both pan and fisheye data). The test and training dataset were disjointed. Table 1 shows that we have highly promising accuracy in both single-query and multi-query settings in terms of Rank-1, Rank-5, and Rank-10. The mAP of 0.853039 for a single query is also good. A quantitative prediction of single camera tracking, pairwise signature ReID, and phase-based signature ReID is achieved by comparing predicted results with the ground truth at frame level or object level. We apply a predefined threshold (e.g., 0.7) to compute matching candidates and pick the highest score for final results. A true positive is a match where the object-signature IDs are the same but the camera IDs are not the same. A false positive occurs where the object-signature IDs are not the same. A false negative occurs if there is no match in both ground truth and matching results. The precision, recall, and F1-score are defined as:

(8) $$TNR=\frac{TN}{TN+FP}=1-FPR$$

(9) $$Precision=\frac{TP}{TP+FP}\;Recall=\frac{TP}{TP+FN}$$

(10) $$F1=2\times \frac{Precision*Recall}{Precision+Recall}.$$

**Figure 10:**
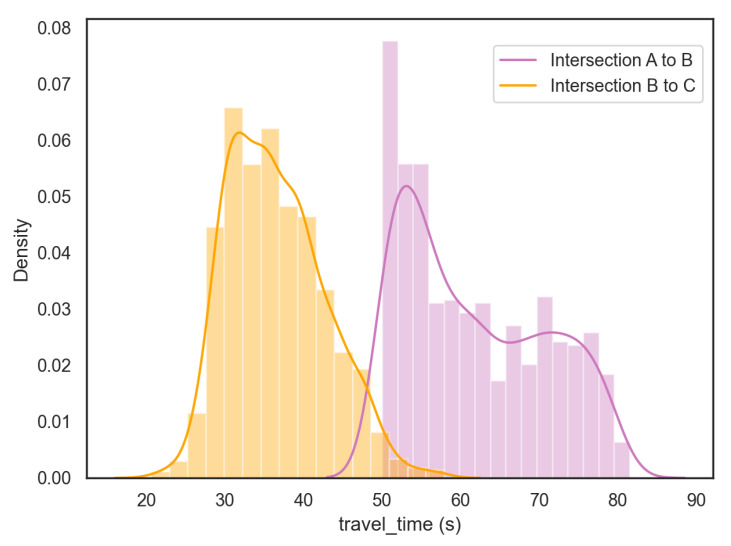
Travel-time distribution results of intersection A to intersection B and intersection B to intersection C.

The evaluation metrics we used for quantitative evaluation include precision, recall, f1-score, and speed for the four major modules of the proposed method. In Table 2, we compare our methods with the state-of-the-art technologies on the VeRi-776 dataset [20] for vehicle ReID. Our approach achieved the best performance at Rank-1 and very competitive performance on mAP. In Table 3, we show that our pipeline has achieved promising performance overall on three modules: single camera tracking, pairwise signature ReID, and phase-based signature ReID. Figure 10 and Table 4 show the travel-time distribution and statistics information of the test set. The distribution of intersection A to B shows two spikes for this road segment, which aligns with our investigation: the first vehicle queued in the lane influences the actual travel time of that group of vehicles. The signature ReID performance in both single query and multi-query settings are shown in Table 1. The ReID model training takes about 2 h on our device (with one NVIDIA Titan V). In Table 5, we present the processing time of each component for a pan camera 5-minute video clip (1280×960, 25 fps)—the overall pipeline costs 221 to 237 s and achieves real-time performance. For a fisheye camera 5-minute video clip (1280×960, 10 fps), the overall pipeline costs 101 to 122 s and also achieves real-time performance.

## 5. Discussions

Although we collected the current dataset as videos of similar background, the dataset has its practical significance on real traffic intersections to handle challenges of robust multi-object multi-camera tracking and accurate travel-time estimation in the presence of factors such as ambiguous vehicles, light and weather conditions, and occlusions. Because our studied intersections are not super large (within five lanes) and do not have many vehicle occlusions, we did not observe a major difference on performance between light and heavy traffic conditions. Therefore, it is encouraged to conduct separate evaluations on different traffic conditions when moving to larger intersections for future work. In addition, for more intersections forming grid networks beyond line segments, it is promising to extend our approach for multiple directions such as left and right turns.

## 6. Conclusions

In this paper, we introduce a novel method and a real-time system to estimate vehicle travel time by leveraging video processing on multiple intersections.

Our key contributions can be summarized broadly as follows: (1) we propose a novel video-based signature ReID method with high precision that plays a key role for multi-camera vehicle tracking; (2) we introduce a novel way to extend pairwise signature matching to phase-based signature matching to tackle the sequential-group-signature ReID problem on traffic video datasets; and (3) we propose a real-time video processing system for multi-camera vehicle tracking and travel-time estimation; We evaluated the proposed framework on a real-world dataset from intersections in Florida with pan and fisheye cameras. Although our results were presented for three intersections, the pairwise intersection nature of our approach allows for scaling this approach to larger numbers of intersections. Overall, our experiments demonstrated the viability, effectiveness, and scalability of the proposed approach. Our future work will extend this to computing origin–destination travel times to all pairs of inputs and outputs across all intersections in a corridor or a traffic network.

## Figures and Tables

**Table 1:** Quantitative evaluation of our discriminator for signature ReID matching.

Component			Single Query (Re-Ranking)				Multi-Query	
**Task**	**Dataset**	**Loss**	**mAP**	**Rank1**	**Rank5**	**Rank10**	**Rank5**	**Rank10**
Signature ReID	Veri	Classification	0.6670	0.9052	0.9344	0.9601	0.8796	0.9464
Signature ReID	Veri	Classification + Verification	0.7173	0.9428	0.9642	0.9780	0.9392	0.9678
Signature ReID	Ours	Classification	0.7938	0.8857	0.8857	0.9214	0.8500	0.8500
Signature ReID	Ours	Classification + Verification	0.8530	1.000	1.0000	1.0000	0.9643	1.0000
ReID (no phase)	Ours	Classification + Verification	0.8526	1.000	1.0000	1.0000	0.9641	1.0000

**Table 2:** Quantitative evaluations of ReID Component. Comparison with the state-of-the-art methods on the VeRI-776 dataset [20].

VeRi-776 Dataset			
**Method**	**Backbones**	**mAP (%)**	**Rank-1 (%)**
GoogLeNet [58]	GoogLeNet	17.81	52.12
AAVER [59]	ResNet-50	58.52	88.68
VANet [60]	GoogLeNet	66.34	89.78
PAMTRI [61]	ResNet-50	71.88	92.86
SAN [62]	ResNet-50	72.5	93.3
Ours	ResNet-50	71.73	94.28

**Table 3:** Quantitative evaluation of proposed method.

Task	Precision	Recall	F1-Score
EAMTT [63]	0.871916	0.786947	0.827255
DeepMOT [64]	0.931207	0.891345	0.910840
Single-Camera Tracking (ours)	0.96002	0.93980	0.94980
Pairwise Signature ReID	1.000000	0.836111	0.910741
Phase-based Signature ReID	1.000000	0.816667	0.899082

**Table 4:** Travel-time computation results.

Intersections	Mean of Travel Times	Standard Deviation
A to B	61.80 s	9.03 s
B to C	36.55 s	6.18 s

**Table 5:** Processing time analysis of proposed method for 5-min video clip (300 s).

Component	CPU/GPU	Processing Time (s)
Single-Camera Tracking	NVIDIA TITAN V	166∼170
Signature ReID	NVIDIA TITAN V	50∼60
Travel Time Computation	CPU (16 cores)	5∼7
Overall Pipeline	GPU + CPU	221∼237

## Data Availability

The data presented in this study are available on request from the corresponding author.
